# Supplementary material for: Shallow coastal zones are key mediators in Arctic land-ocean carbon fluxes
Source: Commun Earth Environ. 2025 Nov 17;6(1):909. doi: 10.1038/s43247-025-02846-5 (PMC12623233; doi:10.1038/s43247-025-02846-5)
Supplement: Supplementary file 2 — Supplementary Information [file 43247_2025_2846_MOESM2_ESM.pdf]

Shallow coastal zones are key mediators in Arctic land-ocean carbon fluxes

F.C.J. van Crimpen[1], L. Madaj[1], J.M. van Genuchten[7], T. Tesi[2], D. Whalen3, K. Scharffenberg[5], L. Bröder[4], M. Fritz[6], J.E. Vonk[1]

[1]Department of Earth sciences, Vrije Universiteit Amsterdam, Amsterdam, The Netherlands.

[2]National Research Council, Institute of Polar Sciences, Bologna, Italy.

[3]Geological Survey of Canada, Natural Resources Canada, Dartmouth, Nova Scotia, Canada.

[4]Geological Institute, Swiss Federal Institute of Technology (ETH), Zurich, Switzerland.

[5] Department of Fisheries and Oceans Canada, Winnipeg, Manitoba, Canada

[6] Department of Periglacial research, Alfred Wegner Institute, Helmholtz Centre for Polar and Marine research, Potsdam, Germany.

[7] Department of Geosciences Centre for ice, Cryosphere, Carbon, and Climate (iC3), The Arctic University of Norway, Tromsø, Norway

Corresponding author: Fleur van Crimpen (f.c.j.van.crimpen@vu.nl)

This supplementary information consist of:

Figure 1: Fractionated samples and their corresponding weight (%), sampling locations are ordered from west (left) to east (right).

Figure 2: Fractionated samples and their corresponding $\delta^{13}C$ (‰), sampling locations are ordered from west (left) to east (right).

Figure 3: Fractionated samples and their C/N ratios, sampling locations are ordered from west (left) to east (right).

Figure 4: Fractionated samples and their OC% in a logarithmic scale, sampling locations are ordered from west (left) to east (right).

Figure 5: Additional Scanning Electron Microscope Images of samples ranging from low density to high density > 200 µm.

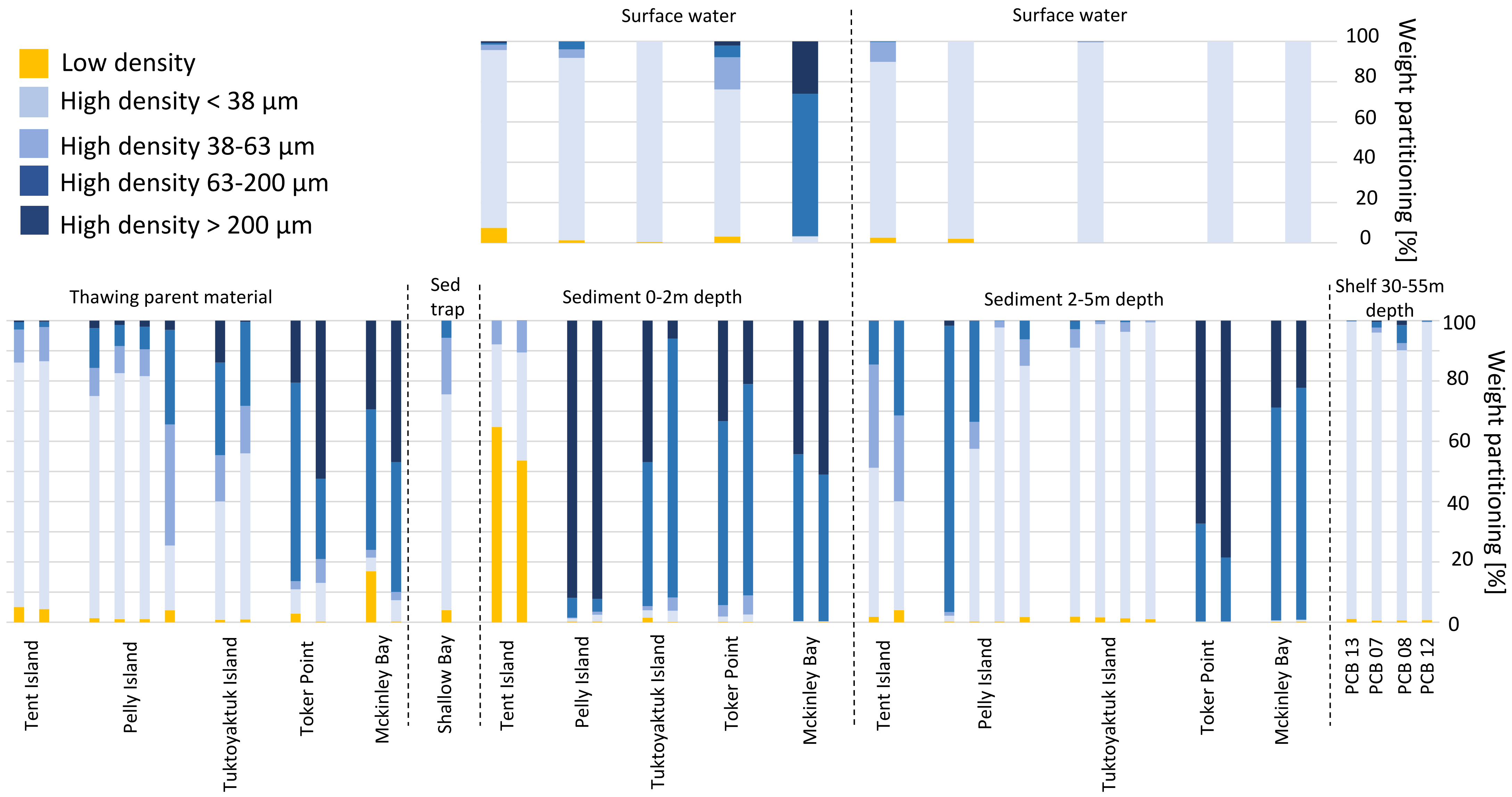

Figure 1: Fractionated samples and their corresponding weight (%), sampling locations are ordered from west (left) to east (right).

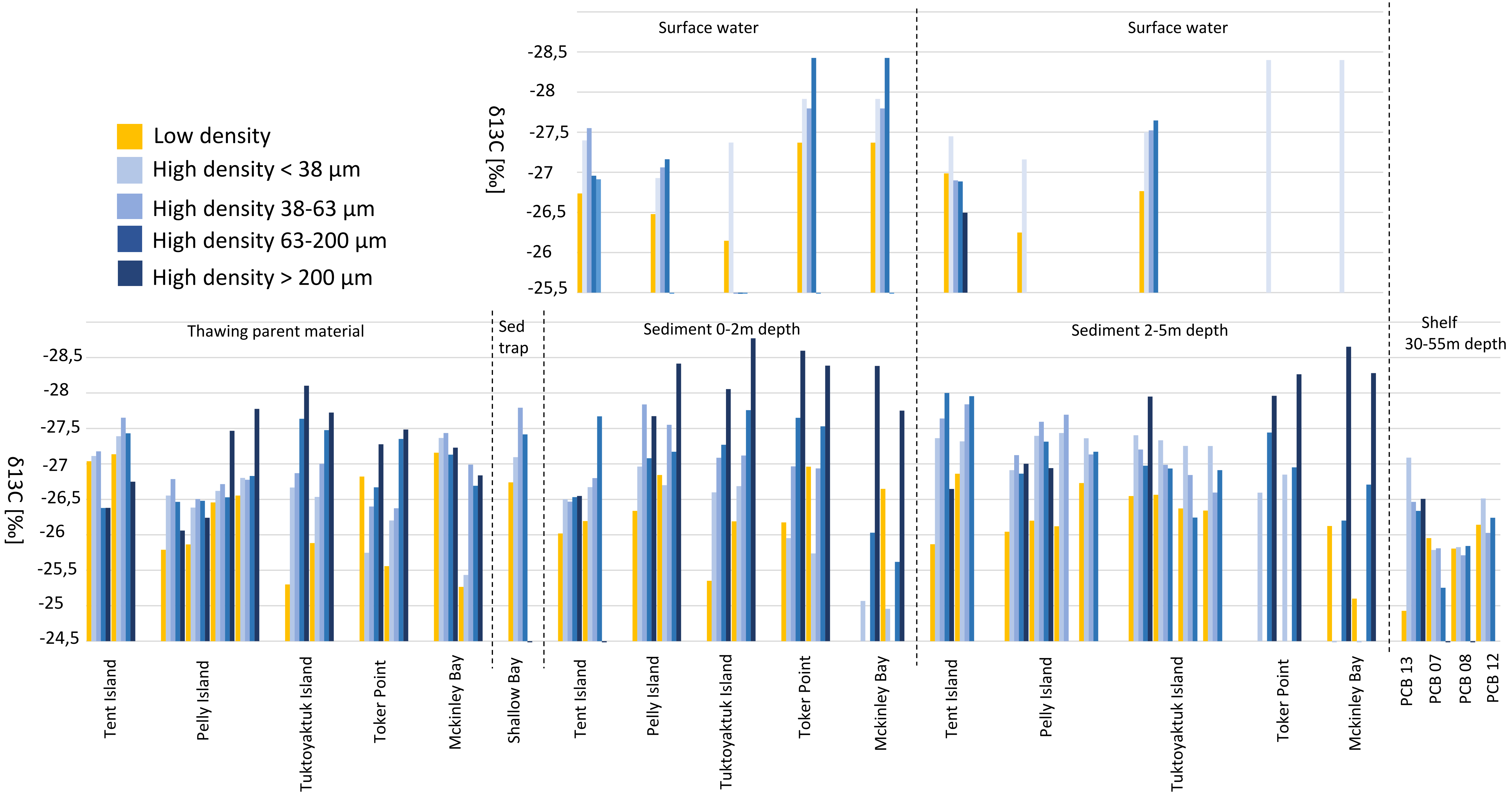

Figure 2: Fractionated samples and their corresponding δ13C (‰), sampling locations are ordered from west (left) to east (right).

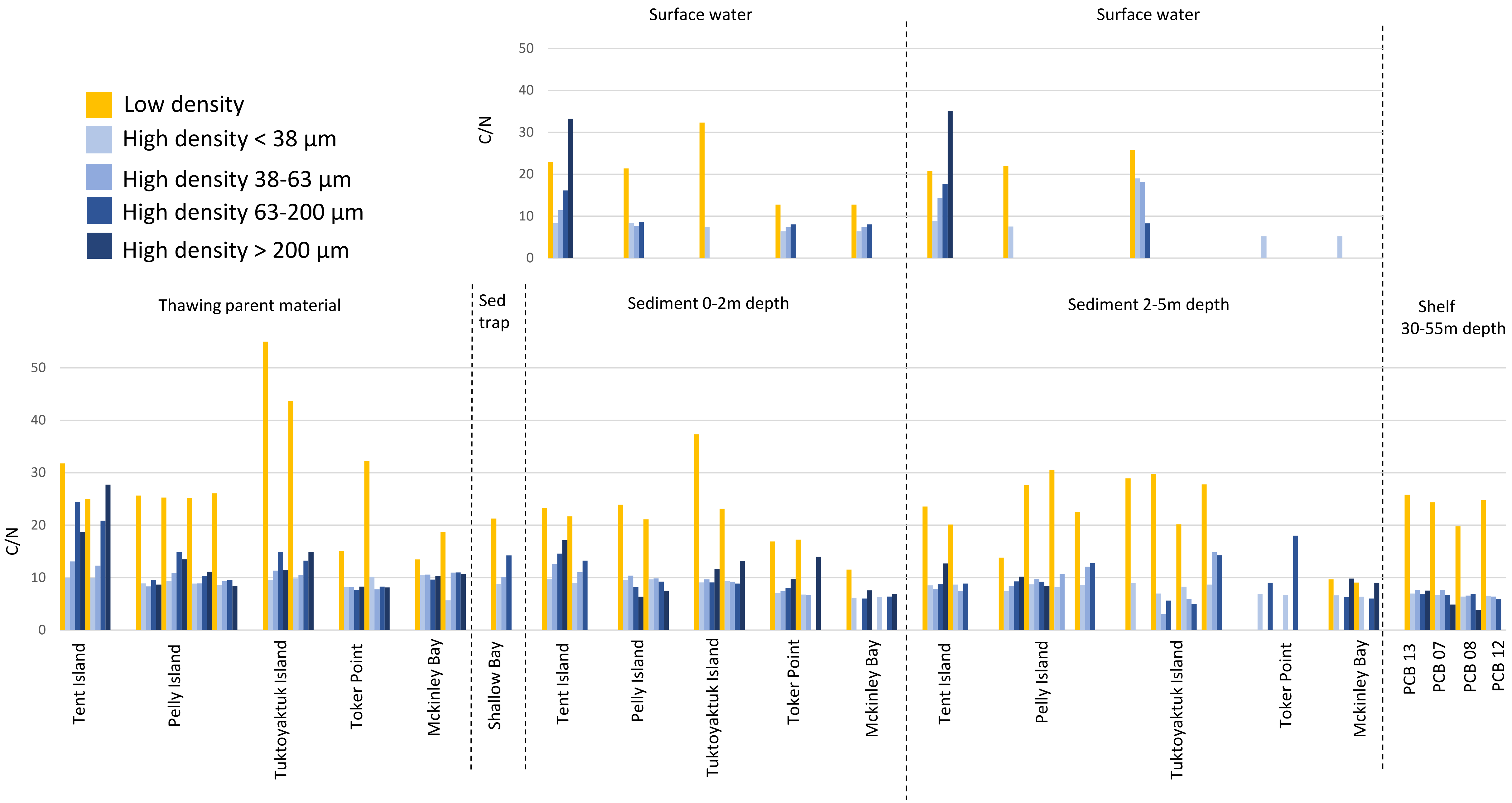

Figure 3: Fractionated samples and their C/N ratios, sampling locations are ordered from west (left) to east (right).

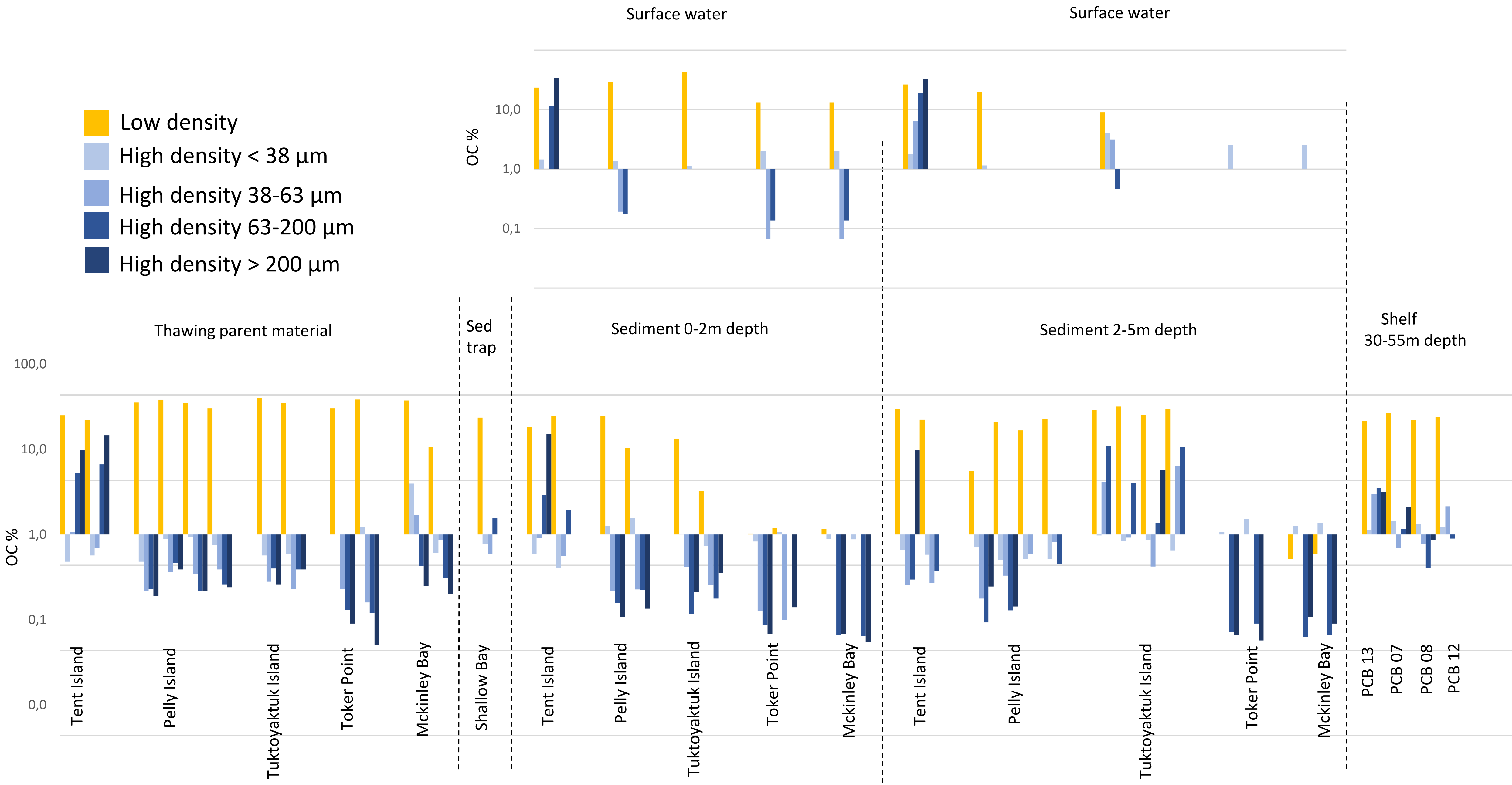

Figure 4: Fractionated samples and their OC% in a logarithmic scale, sampling locations are ordered from west (left) to east (right).

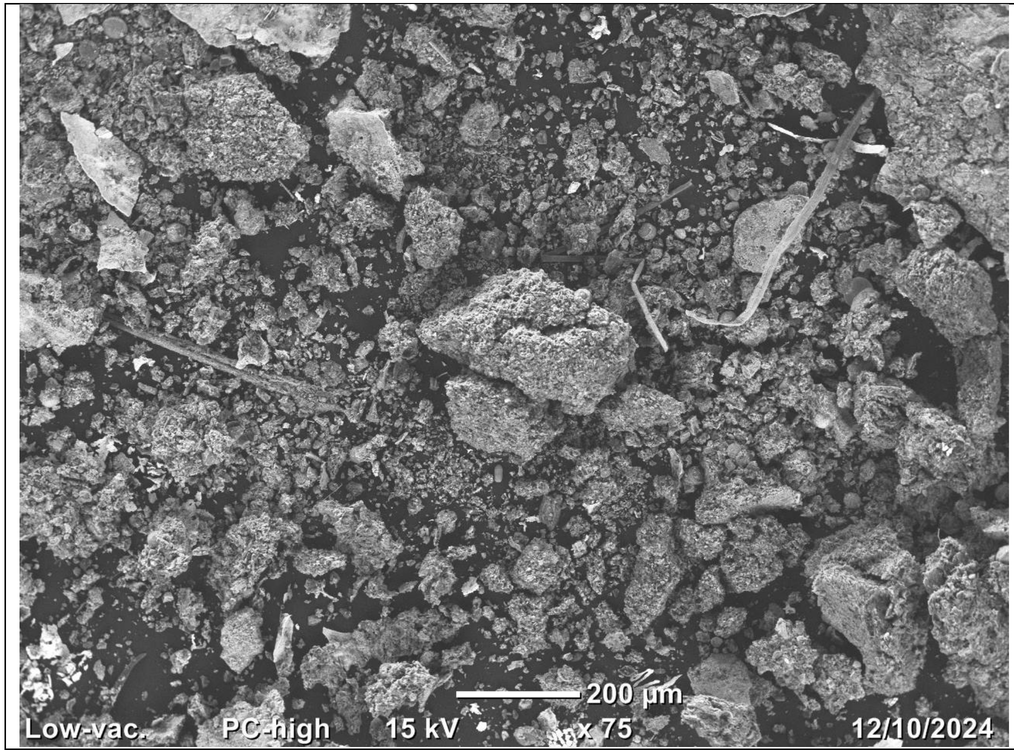	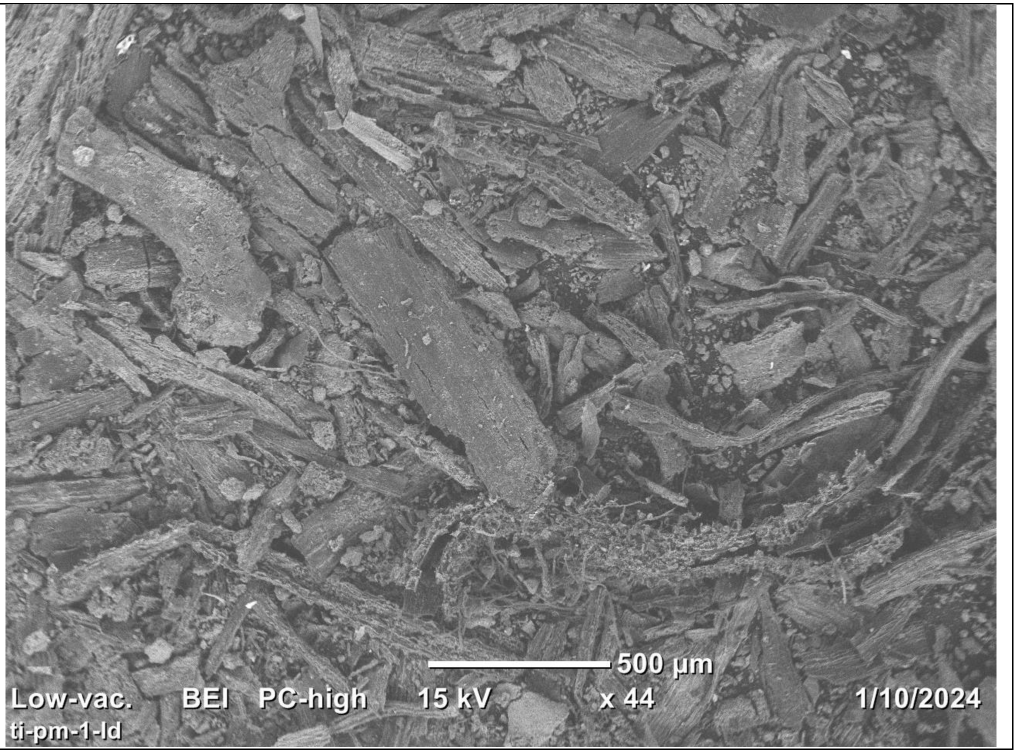
PeCaBeau 08 – surface sediment<br>Low density	Tent Island – parental material<br>Low density

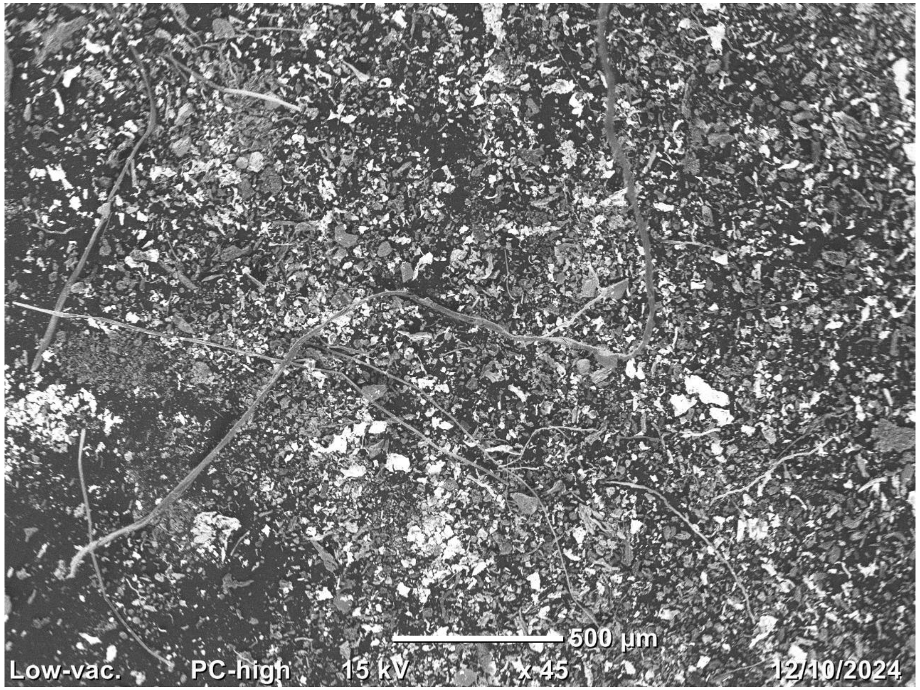	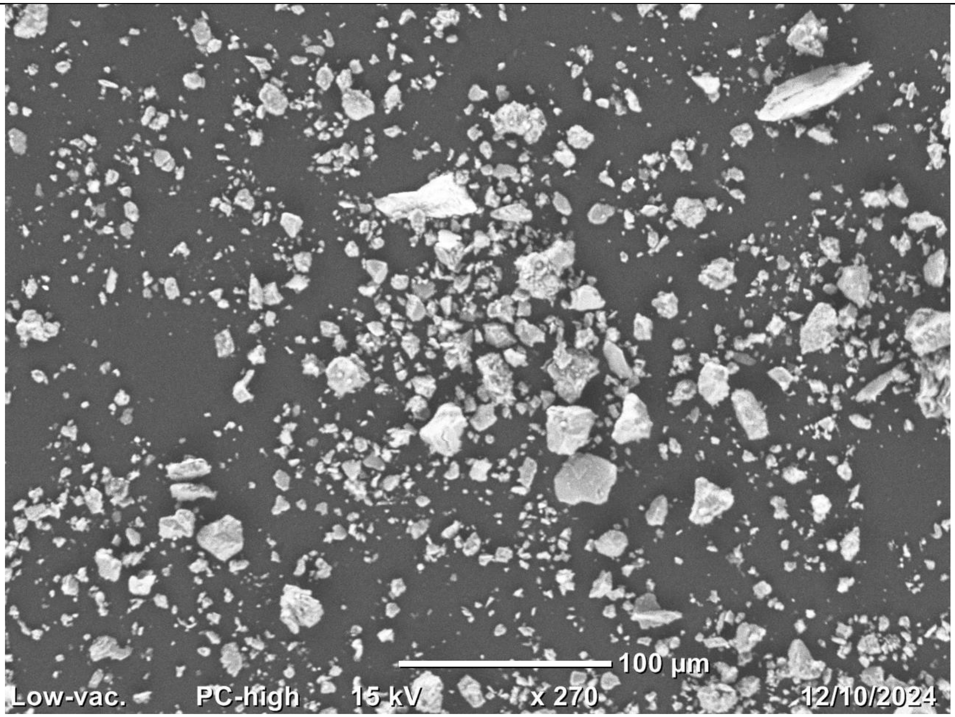
Pelly Island-surface water 0-2 m zone<br>Low density	PeCaBeau 08 – surface sediment<br>High density <38 µm

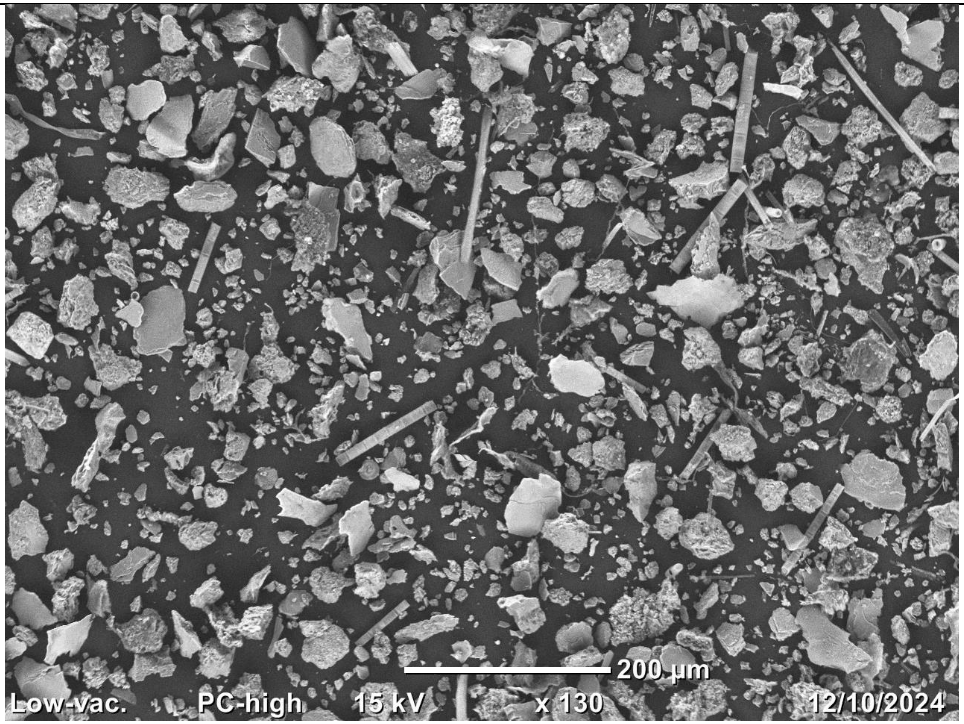

Tuktoyaktuk Island- surface sediment 0-2 m depth
High density 38-63 µm

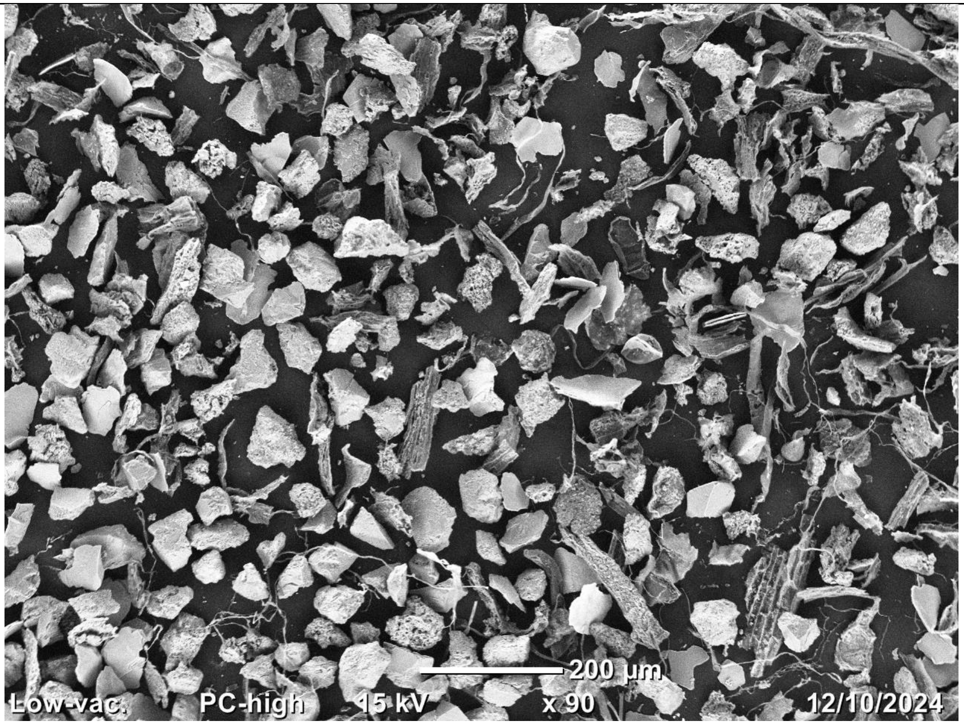

Pelly Island- surface sediment 0-2 m depth
High density 38-63 µm

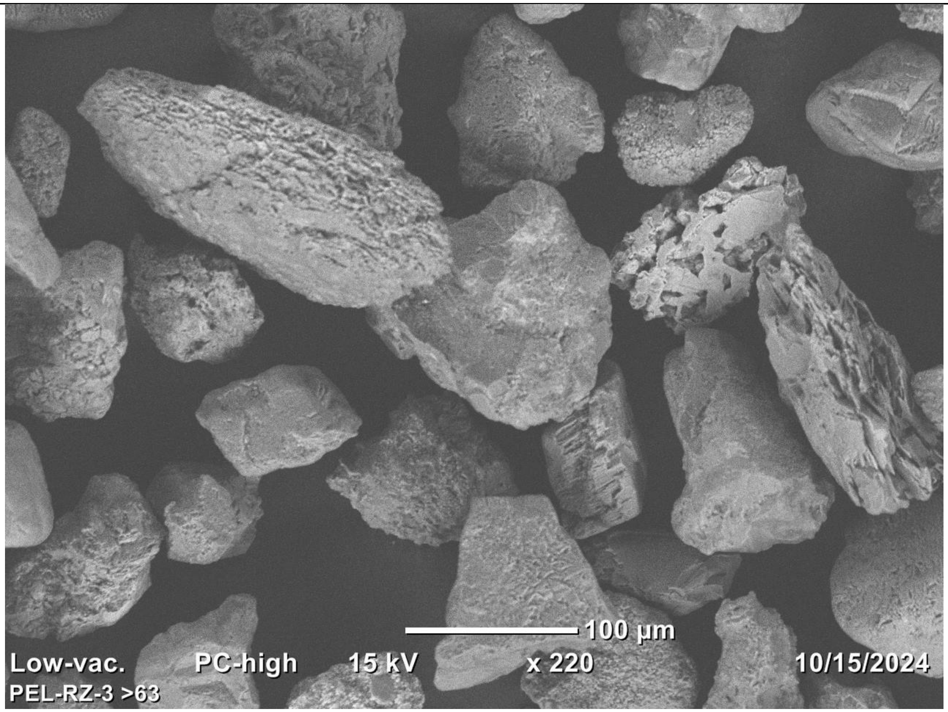	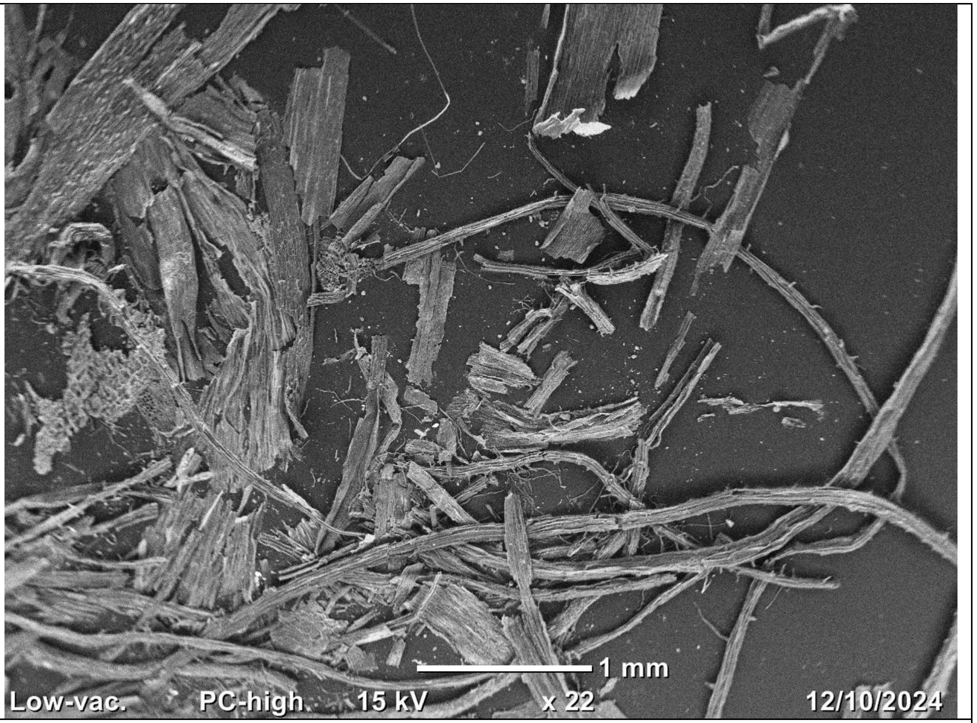
Pelly Island- surface sediment 0-2 m depth<br>High density 63- 200 µm	Tent Island – parental material<br>High density >200 µm

<table>
<tr>
<td>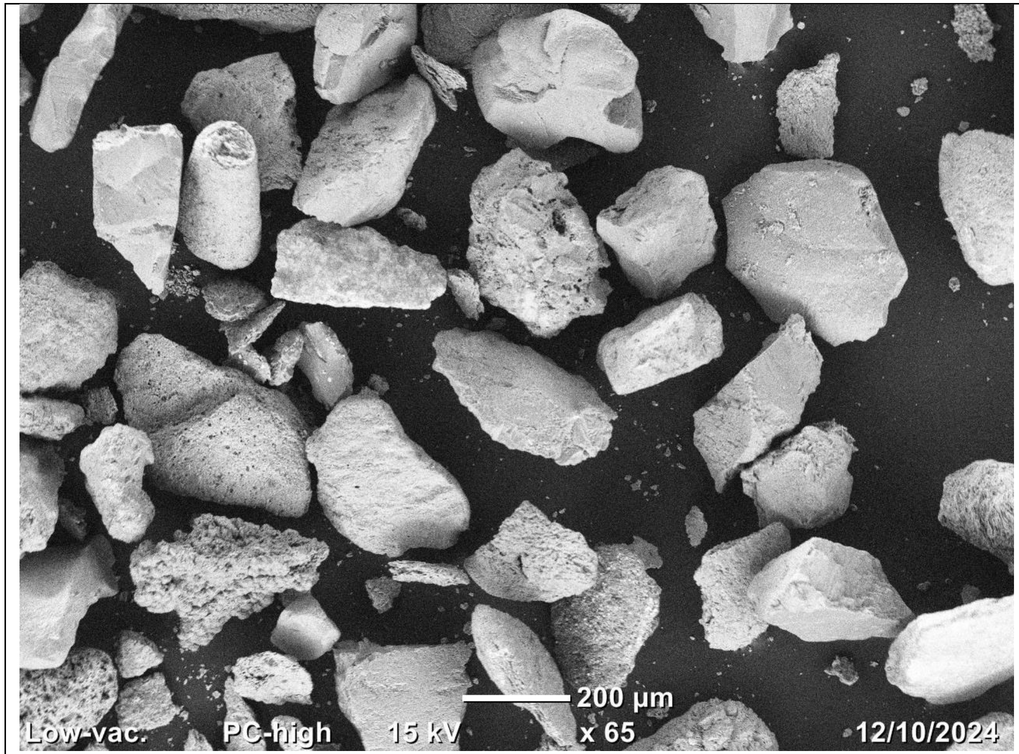
</td>
<td>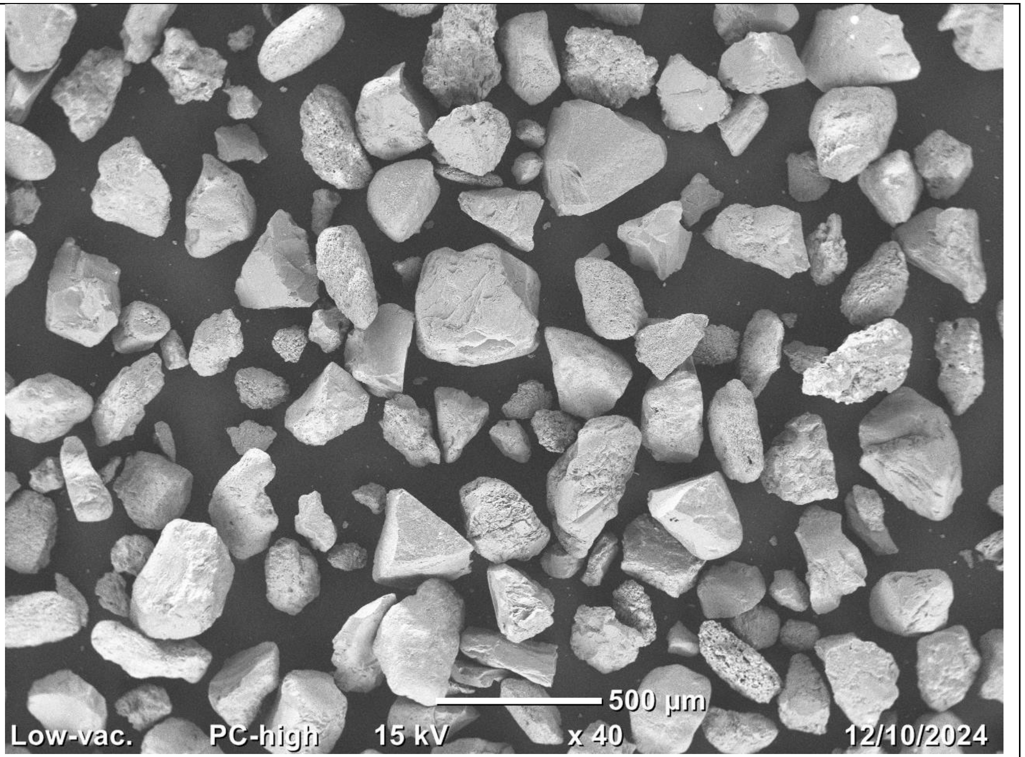
</td>
</tr>
<tr>
<td>Tuktoyaktuk Island- surface sediment 2-5 m depth
High density >200 µm</td>
<td>Tuktoyaktuk Island – parental material
High density >200 µm</td>
</tr>
</table>

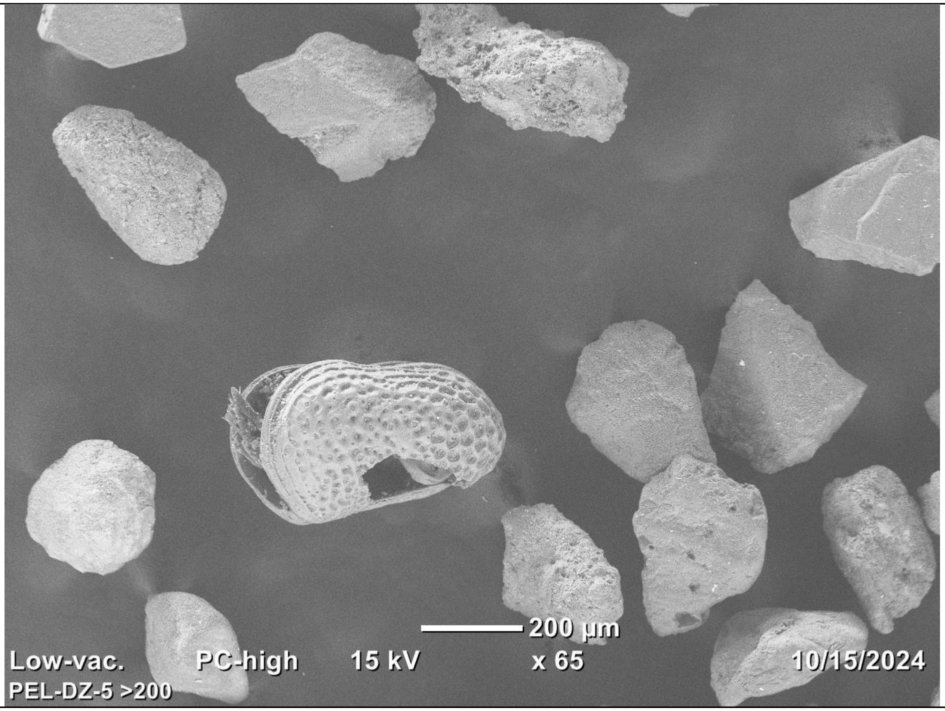

Pelly Island – surface sediment 2-5 m depth
High density >200 µm

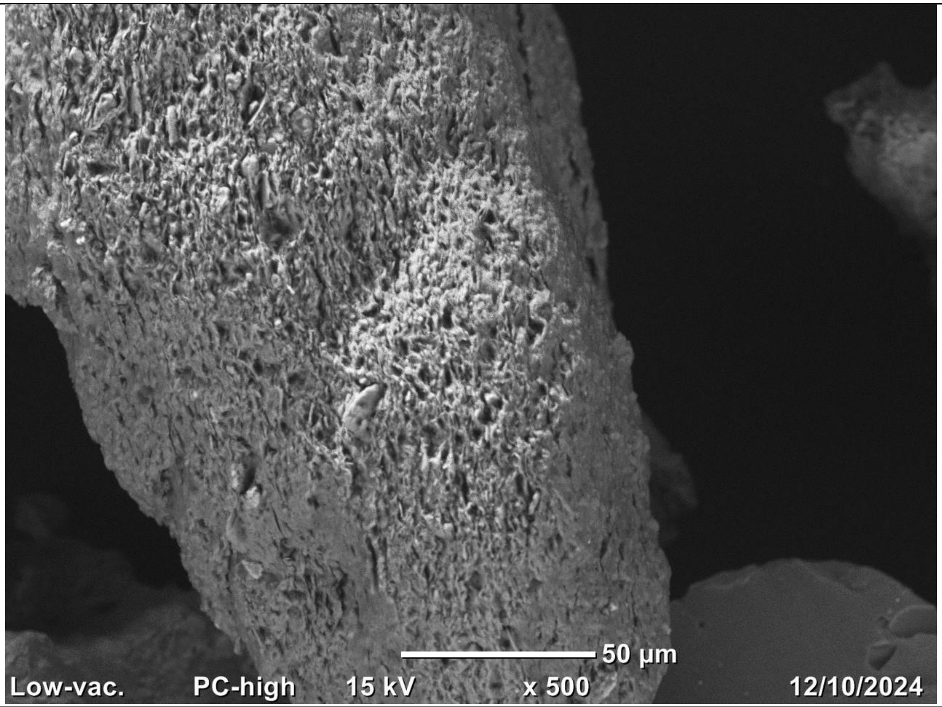

Tuktoyaktuk Island – parent material
High density >200 µm

Figure 5: Additional Scanning Electron Microscope Images of samples ranging from low density to high density > 200 µm.
